# *Martelella alba* sp. nov., isolated from mangrove rhizosphere soil within the Beibu Gulf

**DOI:** 10.1007/s00203-020-02178-2

**Published:** 2021-01-20

**Authors:** Mi Li, Chenghai Gao, Yuyao Feng, Kai Liu, Pei Cao, Yonghong Liu, Xiangxi Yi

**Affiliations:** Institute of Marine Drugs and School of Pharmaceutical Sciences, Guangxi University of Chinese Medicine, NO. 13 Wuhe Road, Nanning, 530200 People’s Republic of China

**Keywords:** *Martelella*, Rhizosphere soil, *Martelella alba* sp. nov

## Abstract

Strain BGMRC 2036^T^ was isolated from rhizosphere soil of *Bruguiear gymnorrhiza* collected from the Beibu Gulf of China. Optimum growth occurred at 28 °C, pH 7.0, and under the conditions of 3–5% (w/v) NaCl. The phylogenetic comparisons of 16S rRNA gene sequences displayed that strain BGMRC 2036^T^ was closely related to *Martelella limonii* NBRC109441^T^ (96.6% sequence similarity), *M. mediterranea* CGMCC 1.12224^T^ (96.5%), *M. lutilitoris* GH2-6^T^ (96.5%), *M. radicis* BM5-7^T^ (96.2%), and *M. mangrove* BM9-1^T^ (95.9%), *M. suaedae* NBRC109440^T^ (95.8%). The phylogenomic tree based on the up-to-date bacterial core gene set indicated that the strain BGMRC 2036^T^ form a clade formed with members of the genera *Martelella*. The major polar lipids include phosphatidylmethylethanolamine, phosphatidylglycerol, phosphatidylcholine, phosphotidylinositol, two unidentified phospholipids, and three unidentified ninhydrin positive phospholipids. The major respiratory quinone is Q-10, which is similar to those of genera *Martelella*. The main cellular fatty acids are C_18:1_ *ω*7*c*, C_16:0_, and C_12:0_ aldehyde. Genome sequencing revealed a genome size of 4.99 Mbp and a G + C content of 62.3 mol%. Pairwise comparison of the genomes of the new strain BGMRC 2036^T^ and the three reference strains *M. endophytica* YC 6887^T^, *M. mediterranea* CGMCC 1.12224^T^, and *M. mangrovi* USBA-857 indicated that gANI value was lower than 81% and a digital DNA–DNA hybridization value was lower than 27%. The strain BGMRC 2036^T^ possessed genes putatively encoding riboflavin synthesis and flavodoxin A polyphasic taxonomic study suggested that strain BGMRC 2036^T^ represented a novel species belonging to the genus *Martelella*, and it was named *Martelella alba* sp. nov. The type strain is BGMRC 2036^T^ (=KCTC 52121^T^ =NBRC 111908^T^).

**Supplementary Information:**

The online version contains supplementary material available at 10.1007/s00203-020-02178-2.

## Introduction

The genus *Martelella* of the family *Aurantimonadaceae* was originally described by Rivas et al. (2005). To date, this genus comprises seven species with validly published names (https://lpsn.dsmz.de/genus/martelella), *M. mediterranea* as the type species, which were isolated from Lake Martel in Mallorca (Rivas et al. 2005). These species were isolated from different sources, including different roots of halophytes, soil from the root of a mangrove, and the water of Lake Martel in Mallorca (Lee 2019; Bibi et al. 2013; Chung et al. 2016; Zhang and Margesin 2014; Rivas et al. 2005; Kim and Lee 2019). In this study, a novel strain BGMRC 2036^T^ was isolated from rhizosphere soil of mangrove plants *B. gymnorrhiza*. Mangroves are woody salt-tolerant plants that grow at tropical and subtropical coastal intertidal zones with high ecological value (Shi et al. 2020).

## Materials and methods

### Bacterial strain and culture condition

During our investigations of microbial biodiversity in mangrove plants, strain BGMRC 2036^T^ was isolated from rhizosphere soil of *B. gymnorrhiza*, collected from the Beibu Gulf of China (21°55′ N, 108°50′ E). The rhizosphere soil was stored in a sterile plastic bottle at 4 °C as soon as it was collected and then transported to the laboratory within 12 h. Soil (2 g) was added to 20 mL sterilized seawater, shaken at 37 °C for 1 h, and then diluted by tenfold. After being incubated at 28 °C for 1 week, 100 μL of the diluent was spread on modified Yeast Malt Extract (modified ISP2; 2.0 g yeast extract, 2.0 g malt extract, 2.0 g d-(+)-glucose anhydrous, 15.0 g agar powder, and 1 L sterilized seawater). A cream colony isolation and purification on modified ISP2 medium was designed a new strain and then stored at − 80 °C with a 20% (v/v) glycerol suspension. Strains *M. suaedae* NBRC109440^T^, *M. limonii* NBRC109441^T^, and *M. mediterranea* CGMCC 1.12224^T^, obtained, respectively, from the National Biological Resource Center, NITE (NBRC) and China General Microbiological Culture Collection Center (CGMCC), were used as references.

### Morphological and physiological characteristics

Morphological and physiological characteristics were observed on modified Yeast Malt Extract modified ISP2 medium unless otherwise stated. Growth and colony morphology were monitored after being induced by continuous incubation over 2 days at 28 °C. A scanning electron microscope (QUANTA 250) was used for the analysis of cell morphology. The presence of strain-flagella was checked by transmission electron microscopy (HT7700; Hitachi, Ltd, Tokyo, Japan) after 2 days of growth on modified ISP2 medium at 28 °C. Cell motility determination was realized by investigating the development process of turbidity throughout a tube using modified ISP2 semisolid medium containing 0.4% agar (Leifson 1960). Gram staining of strain BGMRC 2036^T^ was performed as described by Smibert and Krieg (1994). Oxidase activity was examined using 1% (w/v) *N*, *N*, *N*′, *N*′-tetramethyl-*p*-phenylenediamine reagent, and catalase activity determination was obviously confirmed through bubble production upon the addition of 3% (w/v) hydrogen peroxide (H_2_O_2_) solution (Choi et al. 2014). Sodium chloride (NaCl) requirement and tolerance were tested at 28 °C for 7 days in modified ISP2 liquid medium with NaCl (0‐15%, w/v, with an average interval for 1.0%). The temperature range was determined by incubating cells in modified ISP2 medium broth at 4 °C, 10 °C, 15 °C, 20 °C, 25 °C, 28 °C, 37 °C, 40 °C, and 45 °C for 2 weeks. Growth at different pH values was tested in modified ISP2 liquid medium at 28 °C for 2 weeks (pH 4.0–12.0 at various intervals of 1 pH unit) with the referred buffering system of Xu et al. (2005). As to the colony color determinations, ISCC-NBS color charts were adopted (Kelly and Judd 1965). Hydrogen sulfide production and hydrolysis of substrates (cellulose, gelatin, starch, Tween 20, 40, and 80) were performed according to the description of Tindall et al. (2007). Coagulation and peptonization of milk were investigated according to the method of Gonzalez et al. (1978). Biochemical tests were performed with API ZYM, API 50CH, and API 20E strips (BioMérieux, Marcy-l’Étoile, France) according to the guidance of manufacturer. Utilization of carbon and nitrogen source was studied on Biolog GEN III MicroPlates (Biolog Hayward, CA, USA). The incubation temperature was at 28 °C and the result was monitored after 48 h.

### Chemotaxonomic characterization

Cells of strain BGMRC 2036^T^ and the reference strain were harvested after cultivation on modified ISP2 medium at 28 °C for 3 days, whose polar lipids were resulted by extraction as described by Kamekura (1993), further detection was performed through two-dimensional thin-layer chromatography plates precoated with silica gel 60 GF_254_ (Merck, Kenilworth, NJ, USA) (Minnikin et al. 1984). Menaquinone extraction and analysis were carried out on reversed-phase high-performance liquid chromatography (Komagata and Suzuki 1987; Nakagawa and Yamasato 1993). Cellular fatty acid composition of cell walls was extracted according to Kamekura (1993), analyzed by gas chromatography (G6890N; Agilent Technologies, Inc., Santa Clara, CA, USA), and verified through the Sherlock Microbial Identification System (version 6.0) following the instructions of manufacturer, as reported by Sasser (1990).

### Genomic characterization

The DNA of strain BGMRC 2036^T^ extraction were performed as described by Hoetzinger et al. (2017). The genome was sequenced with Illumina HiSeq 4000 system (Illumina, San Diego, CA, USA) at the Beijing Genomics Institute (Shenzhen, China). The assembly of draft genome was achieved by SOAP denovo software (version 2.04), and the short oligonucleotide of assembling results was subsequently polished by SOAP aligner software (version 2.21) (Li et al. 2008, 2015), details of which are given in Table 3. Average nucleotide identity (ANI) was analyzed with the ANI calculator tool from Ezbiocloud. The digital DNA-DNA hybridization estimate values were based on genome sequence and characterized using formula 2 at the website of Genome-to-Genome Calculator (CGGC) (http://ggdc.dsmz.de/ggdc.php) according to the study of Meier-Kolthoff et al. (2013). The obtained genome sequences were annotated by the NCBI Prokaryotic Genome Annotation Pipeline and for further comparative analyses by rapid annotation using subsystem technology version 2.0. The GenBank accession numbers of BGMRC 2036^T^ and other genus *Martelella* strains are listed in Table 3.

### Phylogeny analysis

The 16S rRNA gene sequence of strain BGMRC 2036^T^ was PCR-amplified with the universal primers 27F and 1492R (Lane 1991) and sequenced using the Sanger method (Zhang et al. 2011). Bacterial DNA extraction and amplification were performed following Li et al. (2007). The 16S rRNA gene sequence similarities were determined using the EzBioCloud database (http://www.ezbiocloud.net) (Niu et al. 2018). Multiple alignments of the sequence profile were performed with Clustal X version 1.83 (Thompson et al. 1997). Phylogenetic trees were constructed through the neighbor-joining (Saitou and Nei 1987), maximum-likelihood (Felsenstein 1981), and Minimum-evolution (Rzhetsky and Nei 1992) algorithms in the MEGA software package (version 7.0) (Kumar et al. 2016). The topology of the phylogenetic tree was reasonably evaluated with bootstrap analysis based on 1000 replicates (Felsenstein 1985). The phylogenomic tree was reconstructed using the up-to-date bacterial core gene set (UBCG v.3) according to its manual (Na et al. 2018).

## Results and discussion

According to API 50CH, strain BGMRC 2036^T^ had different reactions for 198, 8, and 15 of the 49 tested substrates to *M. mediterranea* CGMCC 1.12224^T^, *M. suaedae* NBRC109440^T^, and *M. limonii* NBRC109441^T^, respectively (Table S1). There were 28, 13, and 25 different reactions of the 95 tested substrates (Biolog GEN III MicroPlate) between strains BGMRC 2036 and *M. mediterranea* CGMCC 1.12224^T^, *M. suaedae* NBRC109440^T^, and *M. limonii* NBRC109441^T^ (Table S2). The differences of physiological and biochemical characteristic between strain BGMRC 2036^T^ and its closely related type strains are listed in Table 1 and also mentioned in the species description below.

**Table 1:** Phenotypic characteristics of BGMRC 2036^T^ and closely related species

Characteristic	1	2	3	4	5*	6^§^
Isolation source	Mangrove plants (*Bruguiera gymnorrhiza*)	Water of Lake Martel in Mallorca	Halophyte (*Suaeda maritime*)	Halophyte (*Limonium tetragonum*)	Halophyte (*Carexscabrifolia*)	Soil of the root (mangrove forest)
Temperature range for growth (°C)	25–37 (28)	15–37 (28)	25–45 (28)	25–40 (28)	10–30	15–35
pH range for growth	6–11 (7)	5–12 (7)	5–11 (7)	5–11 (7)	5–10	5–8
NaCl range for growth (%, w/v)	0–8 (3–5)	0–10 (1–4)	3–7 (3)	3–5 (3)	0–11	2–10
Tween 40	+	−	−	−	nd	nd
Polar lipids	PME, PG, PC, PI, 2PL, 3NPL	PME, PE, PI, 2PL, AL	PME, PE, PI, PL, NPL, 3AL, 4L	PME, PE, PI, AL, 2PL	PE, PC, PG, PL, GL, 2L	nd

Strains: 1, *M. alba* BGMRC 2036^T^; 2, *M. mediterranea* CGMCC1.12224^T^; 3, *M. suaedae* NBRC109440^T^; 4, *M. limonii* NBRC109441^T^; 5, *M. caricis* GH2-8^T^; 5, *M. radicis* BM5-7^T^

*PME* phosphatidylmethylethanolamine, *PG* phosphatidyl glycerol, *PE* phosphatidylethanolamine, *PI* phosphatidyl inositol, *PC* phosphatidylcholine, *AL* unidentified ninhydrin positive lipid, *PL* unidentified phospholipid, *NPL* unidentified ninhydrin positive phospholipid, *L* unidentified lipid, *nd* not determined, *+*  positive, *−* negative

*Data taken from (Lee 2019)

^§^Data taken from (Zhang and Margesin 2014)

The major fatty acid of BGMRC 2036^T^ was C_18:1_ *ω*7*c* (48.6%). The remaining fatty acid component (> 10%) included C_16:0_ (22.1%), C_12:0_ aldehyde (14.2%), iso-C_16:1_, and C_14:0_ 3-OH (13.9%), which were similar to that of *M. suaedae* NBRC 109440^T^. However, the minor fatty acids C_16:1_*ω*7*c* and C_16:1_*ω*6*c* were discovered in BGMRC 2036^T^ and were not present in *M. suaedae* NBRC 109440^T^. The fatty acid profile of the new isolate closely resembled those of the type strains of recognized *Martelella* species, although some differences in their proportions were observed. The detailed fatty acid profiles of strain BGMRC 2036^T^ and its related reference strains are shown in Table 2. The major polar lipids consisted of phosphatidylmethylethanolamine, phosphatidylglycerol, phosphatidylcholine, phosphatidyl inositol, two unidentified phospholipids (PL1, PL3), and three unidentified ninhydrin positive phospholipids (NPL1–3) (Fig. S2). The polar lipid profile of BGMRC 2036^T^ was similar to that of the type strains of the genus *Martelella*, with phosphatidylmethylethanolamine and phosphatidyl inositol as the predominant components; phosphatidylglycerol, phosphatidylcholine, one unidentified phospholipid (PL3) and three unidentified ninhydrin positive phospholipids (NPL1–3) were only detected in BGMRC 2036^T^. Furthermore, the absence of phosphatidylethanolamine, one unidentified phospholipid (PL2), seven unidentified ninhydrin positive lipids (AL1–7) and seven unidentified polar lipids (L1–7), along with the presence of phosphatidylglycerol, phospatidyl choline, and two unidentified ninhydrin positive phospholipids (NPL2–3) in the BGMRC 2036^T^ lipid profile helped distinguish the strain from *M. mediterranea*, *M. suaedae*, and *M. limonii* (Fig. S2). Hence, from the data obtained above, strain BGMRC 2036^T^ could clearly be differentiated from its closest phylogenetic relatives. The menaquinone was ubiquinone Q-10, which was identical to that of the *Martelella* genus.

**Table 2:** Cellular fatty acid compositions of strains BGMRC 2036^T^ and related strains

Fatty acid (%)	1	2	3	4	5^§^
Straight-chain saturated					
C_16:0_	**22.1**	**11.2**	**12.4**	**13.5**	nd
C_18:0_	5.9	6.3	7.9	5.4	7.6
C_18:0_ 2-OH	nd	0.3	1.2	1.9	nd
C_18:0_ 3-OH	0.2	1.1	0.2	0.68	nd
Monounsaturated					
C_19:0_ cyclo *ω*8*c*	5.5	**52.3**	6.0	**9.7**	**24.9**
11-methyl C_18:1_*ω*7*c*	0.5	4.6	5.8	5.6	6.8
10-methyl C_19:0_	0.1	1.7	nd	nd	nd
Summed feature 2^ǂ^	**14.2**	**12.1**	**13.8**	7.2	12.4
Summed feature 3^ǂ^	1.7	0.4	0.6	0.7	nd
Summed feature 8^ǂ^	**48.6**	7.3	**50.7**	**52.7**	**41.7**

Strains: 1, *M. alba* BGMRC 2036^T^; 2, *M. mediterranea* CGMCC1.12224^T^; 3, *M. suaedae* NBRC109440^T^; 4, *M. limonii* NBRC109441^T^; 5, *M. radicis* BM5-7^T^. All strains were grown on ISP2 agar. The major fatty acids (greater than 10%) are shown in bold. All data are from this study

*nd* not determined

^ǂ^Summed feature 2 contains iso I-C_16:1_ and/or C_14:0_ 3-OH; summed feature 3 contains C_16:1_*ω*7*c* and/or C_16:1_*ω*6*c*; summed feature 8 contains C_18:1_*ω*7*c* and/or C_18:1_*ω*6*c*

^§^Data taken from (Zhang and Margesin 2014)

**Fig. 1:**
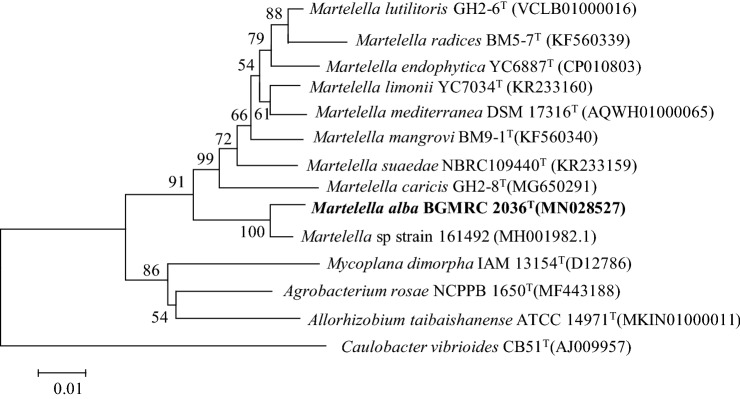
Neighbor-joining phylogenetic tree, based on 16S rRNA gene sequences, shows the position of the BGMRC 2036^T^ with related taxa. The sequence of *Caulobacter vibrioides* CB51^T^ was used as an out group. Asterisks indicate that the corresponding branches were also recovered in trees generated with the maximum-likelihood and maximum-parsimony methods. Numbers at nodes indicated the percentage of 1000 bootstrap replicates. Only bootstrap values above 50% are shown. Bar, 0.01 substitutions per nucleotide position

**Fig. 2:**
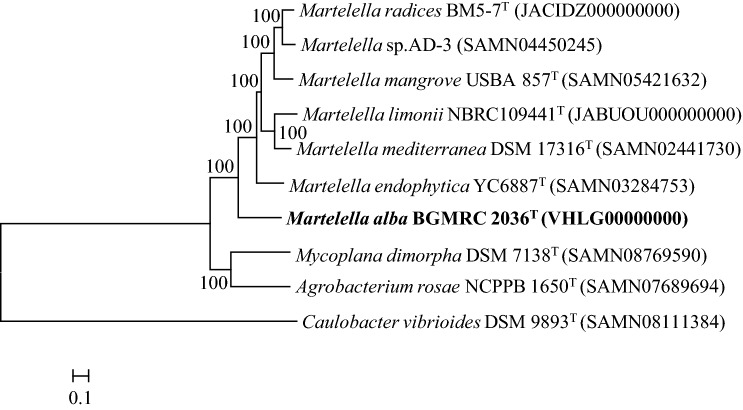
Whole-genome based phylogenetic tree were constructed using UBCGs (concatenated alignment of 92 core genes) and showing the phylogenetic relationship of BGMRC 2036^T^ with reference species in the genus *Martelella*. Gene support indices (GSIs) is given at branching points. Bar, 0.5 substitution per position

The global alignment based on 16S rRNA gene sequence in the EzBioCloud database demonstrated that strain BGMRC 2036^T^ was closely related to *M. limonii* NBRC 109441^T^ (96.6% sequence similarity), *M. mediterranea* CGMCC1.12224^T^ (96.5%), *M. radicis* BM5-7^T^ (96.2%), and *M. suaedae* NBRC109440^T^ (95.9%). Phylogenetic analysis based on the neighbor-joining algorithm, maximum-likelihood algorithm, and minimum-evolution methods revealed that strain BGMRC 2036^T^ formed a clade within members of the genus *Martelella* related to the family *Aurantimonadaceae* (Figs. 1, S3, S4). The phylogenomic tree based on the up-to-date bacterial core gene set also indicated that the strain BGMRC 2036^T^ formed a robust clade within genus *Martelella* (Fig. 2), supporting that strain BGMRC 2036^T^ is a novel species of the genus *Martelella* in agreement with the results of the 16S rRNA gene phylogenic analysis.

The genome size of strain BGMRC 2036^T^ was 4.99 Mbp, and that of N50 was 243,156 base pairs. A total of 71 contigs were obtained (Table 3). The genome sizes of the other three reference strains *M. endophytica* YC6887^T^, *M. mediterranea* CGMCC 1.12224^T^, and *M. mangrovi* USBA-857 were 4.82 Mbp, 5.69 Mbp, 4.63 Mbp, respectively (Table 3). All strains had relatively high G + C contents of more than 60 mol% (Table 3). The G + C content of strain BGMRC 2036^T^ was 62.3 mol%, which was lower than that of *M. limonii* NBRC 109441^T^ and higher than that of other closely related species shown in Tables 1 and 3. The genome orthoANI value between strain BGMRC 2036^T^ and *M. endophytica* YC6887^T^, *M. mediterranea* CGMCC 1.12224^T^, *M. mangrovi* USBA-857 was lower than 81% and a digital DNA-DNA hybridization value was lower than 27% (Table 3). These values were considerably lower than the recommendation of a threshold value of 96% ANI and 70% DNA-DNA relatedness as to the general species definition, indicating that the strain BGMRC 2036^T^ does not attach to *M. mediterranea* and may represent a novel species.

**Table 3:** Genome characteristics of strain BGMRC 2036^T^ and other species of the genus *Martelella*

Characteristic	1	2	3	4	5	6	7	8
Number of scaffolds	55	40	1	4	46	3	18	42
N50 value (Mbp)	0.24	0.39	4.82	4.67	0.24	4.56	4.02	0.24
Genome size (Mbp)	4.99	4.54	4.82	5.69	4.63	5.04	4.45	4.98
G + C content (mol%)	62.3	61.6	62.1	62.4	60.3	62.3	62	59.7
ANI (%)	100	75.5	76.2	75.6	75.9	76.0	76.0	76.1
DDH (%)	100	22.6	22.8	26.9	22.7	23.2	23.4	20.7
DDBJ/EMBL/GenBank accession number	VHLG00000000	JABUOU000000000	CP010803	CP020330	GCA_003001975.1	AYGY00000000	VCLB00000000	JACIDZ000000000

Strains: 1, *M. alba* BGMRC 2036^T^; 2, *M. limonii* NBRC109441^T^; 3, *M. endophytica* YC6887^T^; 4, *M. mediterranea* CGMCC1.12224^T^; 5, *M. mangrovi* USBA-857; 6, *Martelella* sp AD-3; 7, *M. lutilitoris* GH2-6^T^; 8, *M. radicis* BM5-7^T^

The gene content of strain BGMRC 2036^T^ and seven closely related species showed interesting pattern (Table 4). All strains except *M. mediterranea* and *M. mangrovi* encompassed genes putatively encoding flavodoxin and a gene cluster participating in ammonia assimilation (Table 4). Concerning the ABC-type transport systems, toxin–antitoxin replicon stabilization systems and copper transport systems of the seven strains showed different patterns. All strains except *M. endophytica* possessed a gene cluster participating in choline and betaine uptake and betaine biosynthesis. All strains possessed a riboflavin synthesis gene cluster that can produce 5′-phosphate decarboxylase. The strain AD-3 had been reported to possess a high phenanthrene biodegradability, which may have potential for bioremediation of PAH-contaminated hypersaline sites (Feng et al. 2012). Strain BGMRC 2036^T^ possessed a gene cluster participating in nitrogen fixation. In addition, the related strains *Martelella* sp. strain 161,492 (MH001982) is a diazotroph resource in mangrove sediment, which may have relationship with the habitat of mangroves plants (Liu et al. 2020). Thus, new strain may affect mangrove ecosystems and relate to nitrogen fixation in mangrove sediment.

**Table 4:** Comparison of the presence and absence of selected genes in strain BGMRC 2036^T^ and other species of the genus *Martelella*

Genes putatively encoding	1	2	3	4	5	6	7	8
ABC-type transporter dipeptide and oligopeptide	+	−	−	−	+	+	+	−
Toxin–antitoxin replicon stabilization systems	+	+	−	−	−	+	+	−
Copper transport system	−	+	+	−	−	+	+	−
Biogenesis of c-type cytochromes	+	+	−	−	−	+	+	+
Nitrogen fixation	+	−	+	−	−	−	−	+
Cyanate hydrolysis	+	−	−	−	−	−	−	+
Ammonia assimilation	+	+	+	−	−	+	+	+
Superoxide dismutase	−	−	+	+	+	+	+	−
Nitrite reductase	−	−	+	+	+	−	−	−
Choline and betaine uptake and betaine biosynthesis	+	+	−	+	+	+	+	−
Riboflavin synthesis cluster	+	+	+	+	+	+	+	+
Flavodoxin	+	+	+	−	−	+	+	−

Strains: 1, *M. alba* BGMRC 2036^T^; 2, *M. limonii* NBRC109441^T^; 3, *M. endophytica* YC6887^T^; 4, *M. mediterranea* CGMCC1.12224^T^; 5, *M. mangrovi* USBA-8576; 6, *Martelella* sp AD-3; 7, *M. lutilitoris* GH2-6^T^; 8, *M. radicis* BM5-7^T^

### Description of *Martelella alba* sp. nov.

*Martelella alba* (al’ba. L. fem. adj. *alba* white, referring to the color of the colonies).

The Gram-negative, non-motile, and rod-shaped bacteria cells are 0.3–0.4 μm in width and 0.6–1.0 μm in length. Colonies were moist, circular, smooth, white, and 0.1–0.5 mm in diameter after being maintained on modified ISP2 agar at 28 °C for 2 days. Growth occurred at 25–37 °C (optimum, 28 °C) with pH range 6.0–11.0 (pH 7.0) and containing 0–8.0% (w/v) NaCl (3–5%). The strain was negative for nitrate reduction, hydrolysis of gelatin, cellulose, starch, Tween 20, 40, and 80, and milk coagulation and peptonization. In the API 20E, *O*-nitrophenyl-*β*-d-galactopyranoside, VP test, glucose fermentation, glucose fermentation, amygdalin, and arabinose were positive. In the API ZYM, alkaline phosphatase, esterase (C4), leucine arylamidase, yaline arylamidase, acid phosphatase, naphthol-ASBI-phosphohydrolase, *β*-galactosidase, *α*-glucanase, *β*-glucosidase, and *N*-acetyl-*β*-glucosaminidase activities were positive. The major fatty acids of strain BGMRC 2036^T^ were C_18:1_ *ω*7*c* and C_16:0_, while ubiquinone Q-10 was found to be the predominant menaquinone. The main polar lipids included phosphatidylmethylethanolamine, phosphatidylglycerol, phosphatidylcholine, phosphatidyl inositol, two unidentified phospholipid (PL1 and PL3), and three unidentified ninhydrin positive phospholipid (NPL1-3). This strain type was BGMRC 2036^T^ (=KCTC 52121^T^ =NBRC 111908^T^) isolated from the rhizosphere soil of *B. gymnorrhiza* from the Beibu Gulf.

## Supplementary Information

Below is the link to the electronic supplementary material.

Supplementary file1 (DOCX 2968 KB)

## Abbreviations

KCTC: The Korean Collection for Type Cultures
MCCC: The Marine Culture Collection of China
Q: Ubiquinone
PME: Phosphatidylmethylethanolamine
PG: Phosphatidylglycerol
PE: Phosphatidylethanolamine
PI: Phosphatidyl inositol
PC: Phosphatidyl choline
AL: Unidentified aminolipid
NPL: Unidentified ninhydrin positive phospholipid
PL: Unidentified phospholipid
L: Unidentified lipid
