# Supplementary material for: Martelella alba sp. nov., isolated from mangrove rhizosphere soil within the Beibu Gulf
Source: Arch Microbiol. 2021 Jan 20;203(4):1779–86. doi: 10.1007/s00203-020-02178-2 (PMC8055630; doi:10.1007/s00203-020-02178-2)
Supplement: Supplementary file 1 — Supplementary file1 (DOCX 2968 KB) [file 203_2020_2178_MOESM1_ESM.docx]

***Martelella alba* sp. nov.*,* isolated from** **mangrove rhizosphere soil within the Beibu Gulf**

Mi Li, Chenghai Gao, Yuyao Feng, Kai Liu, Pei Cao, Yonghong Liu*, Xiangxi Yi*

Institute of Marine Drugs/School of Pharmaceutical Sciences, Guangxi University of Chinese Medicine, NO. 13 Wuhe Rood, Nanning 530200, People’s Republic of China

***Author for correspondence:** Xiangxi Yi, E-mail: [yixiangxi2017@163.com](mailto:yixiangxi2017@163.com)

Yonghong Liu, E-mail: yonghongliu@scsio.ac.cn

**Subjective category:** Alphaproteobacteria

**Running title:** *Martelella alba* sp. nov.

**Supplementary Materials**

**Table S1**. Utilization of (API 20E), enzyme activities (API ZYM) and Acid produced from (API 50CH) of BGMRC 2036^T^ and closely related species.

Strains: 1, *M. alba* BGMRC 2036^T^; 2, *M. mediterranea* CGMCC1.12224^T^; 3, *M. suaedae* NBRC109440^T^; 4, *M. limonii* NBRC109441^T^. +, positive; -, negative, nd, not determined.

| **Characteristic** | **1** | **2** | **3**§ | **4**φ |
| --- | --- | --- | --- | --- |
| **Utilization of (API 20E):** |  |  |  |  |
| Citrate utilization test | - | + | - | + |
| Urease test | - | + | + | - |
| VP test | + | - | - | - |
| Glucose fermentation | + | - | + | - |
| Mannitol fermentation | - | - | + | - |
| Rhamnose | - | w | - | - |
| Sucrose fermentation | + | + | + | - |
| Melibiose | w+ | W | + | - |
| Amygdalin | + | + | + | - |
| Oxidase | - | + | - | - |
| NO_2_ | - | + | - | - |
| N_2_ | - | - | + | - |
| **Enzyme activities (API ZYM)**: |  |  |  |  |
| Alkaline phosphatase | + | + | - | - |
| Esterase lipase (C8) | - | + | - | - |
| Yaline arylamidase | w+ | - | w+ | - |
| Cystine arylamidase | - | - | w+ | - |
| Acid phosphatase | + | - | + | w+ |
| *β*-Galactosidase | + | - | + | - |
| *β*-Glucosidase | + | + | + | - |
| *N*-Acetyl-*β*-glucosaminidase | + | + | - | - |
| **Acid produced from (API 50CH)** |  |  |  |  |
| D-Mannose | - | + | - | - |
| Meso-erythritol | - | + | - | - |
| D-Arabinose | - | + | + | - |
| L-Arabinose | + | + | + | - |
| D-Ribose | + | + | + | - |
| D-Xylose | / | + | + | - |
| L-Xylose | - | + | - | - |
| D-Adonitol | - | + | - | - |
| Methyl–*β*-D-pyrangioside | - | + | + | - |
| D-Galactose | + | + | + | - |
| *α*-D-Glucose | + | + | + | - |
| D-Fructose | + | + | + | - |
| D-Mannose | + | + | + | - |
| Myo-inositol | - | + | - | - |
| D-Mannitol | - | + | - | - |
| D-Sorbitol | - | + | - | - |
| methyl *α*-D-glucopyranoside | + | - | + | - |
| Arbutine | + | - | - | - |
| Salicin | + | - | + | - |
| D-Cellobiose | + | + | + | - |
| D-Maltose | + | + | + | - |
| D-Lactose | - | + | + | - |
| D-Melibiose | - | + | + | - |
| D-Sucrose | + | + | + | - |
| D-Trehalose | + | + | + | - |
| D-Raffinose | - | + | + | - |
| D-Turanose | + | + | + | - |
| D-Lyxose | + | + | + | - |
| D-Tagatose | - | + | - | - |
| D-Fucose | - | + | - | - |
| L-Fucose | - | + | + | - |
| D-Arabinitol | / | + | + | - |
| L-Arabinitol | / | - | - | - |
| 2-Ketogluconate | - | - | + | - |

**Table S2**. BIOLOG GEN Ⅲ MicroPlate of BGMRC 2036^T^ and closely related species.

Strains: 1, *M. alba* BGMRC 2036^T^; 2, *M. mediterranea* CGMCC1.12224^T^; 3, *M. suaedae* NBRC109440^T^; 4, *M. limonii* NBRC109441^T^. +, positive; -, negative, nd, not determined.

| **Characteristic** | **1** | **2** | **3** | **4** |
| --- | --- | --- | --- | --- |
| Dextrin | - | + | + | - |
| Stachyose | + | - | - | + |
| D-Salicin | + | + | + | - |
| *N*-Acetyl-D-glucosamine | - | + | - | - |
| *N*-Acetyl-*β*-D-mannosamine | + | + | - | - |
| *N*-Acetyl-D-galactosamine | - | + | - | - |
| 4% NaCl | + | + | + | - |
| 8% NaCl | + | + | + | - |
| D-Fructose | + | - | + | + |
| L-Rhamnose | + | + | + | - |
| 1 % Sodium lactate | + | + | + | - |
| Fusidic acid | - | + | + | - |
| D-Sorbitol | + | + | - | + |
| Myo-inositol | - | + | - | + |
| Troleandomycin | + | + | + | - |
| Minocycline | - | + | + | - |
| Glycyl-L-proline | - | + | + | - |
| L-Alanine | - | + | - | + |
| L-Glutamic acid | + | - | + | - |
| D-Galacturonic acid | + | + | + | - |
| L-Galactonic acid lactone | + | - | + | - |
| D-Gluconic acid | - | + | - | - |
| D-Glucuronic acid | + | - | + | + |
| Glucuronamide | - | + | - | - |
| Mucic acid | - | + | - | - |
| Quinic acid | - | + | - | - |
| Vancomycin | + | + | + | - |
| Tetrazolium blue | + | + | - | - |
| Methyl pyruvate | + | + | + | - |
| Citric acid | - | + | - | + |
| *α*-Keto-glutaric acid | + | - | - | + |
| D-Malic acid | - | + | + | + |
| L-Malic acid | - | + | + | + |
| Bromo succinic acid | - | + | - | - |
| Lithium chloride | + | + | + | - |
| Potassium tellurite | - | - | + | - |
| Tween 40 | - | + | - | + |
| *γ*-Amino-butryric acid | - | + | - | + |
| *α*-Hydroxy-butyric acid | - | + | - | + |
| Acetoacetic acid | - | + | - | + |
| Propionic acid | - | - | + | - |
| Formic acid | - | + | - | + |
| Aztreonam | + | + | + | + |
| Sodium butyrate | + | - | + | - |


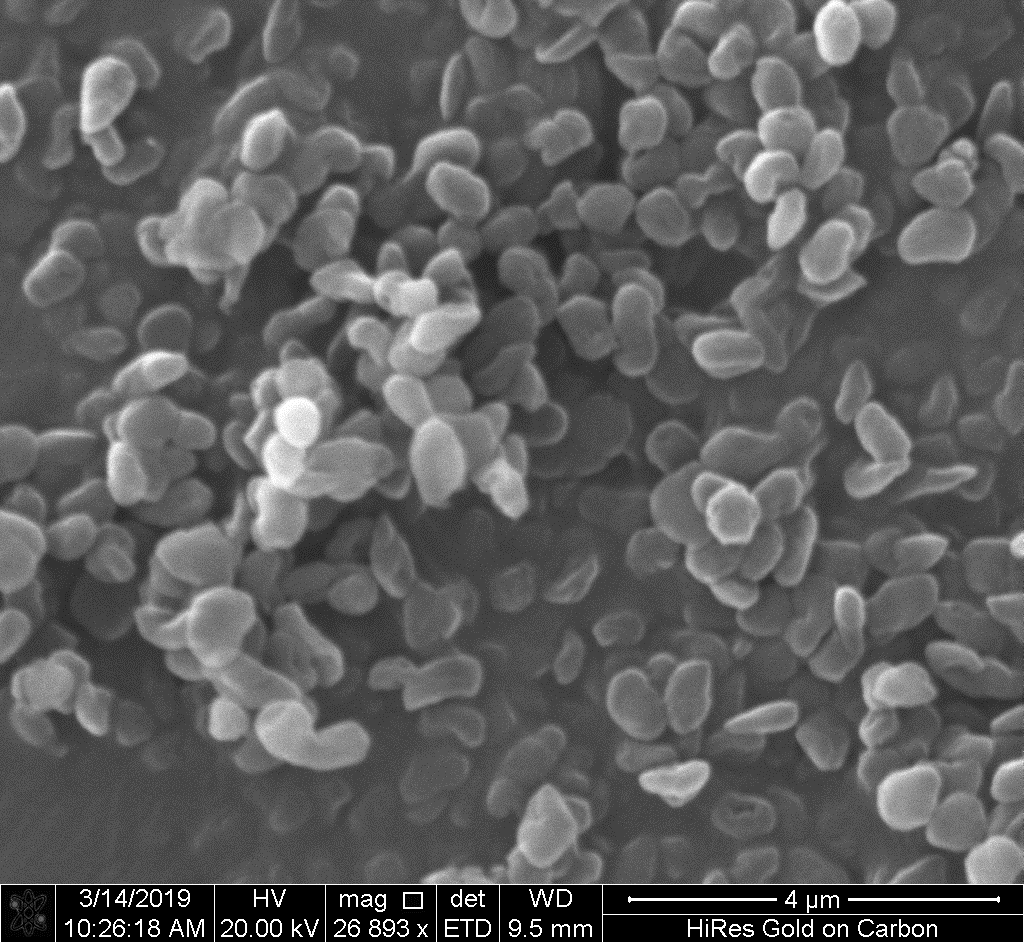

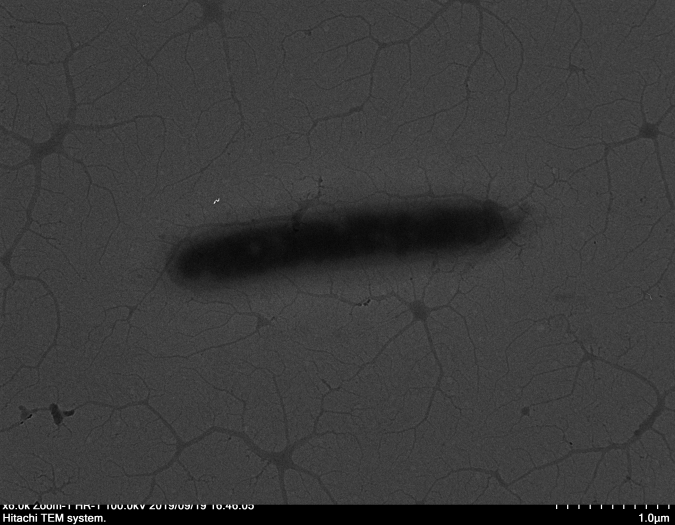


**Fig. S1.** Scanning electron microscopy and transmission electron micrograph of cells of strain BGMRC 2036^T^ grown on ISP2 agar for 2 days at 28 °C.

**
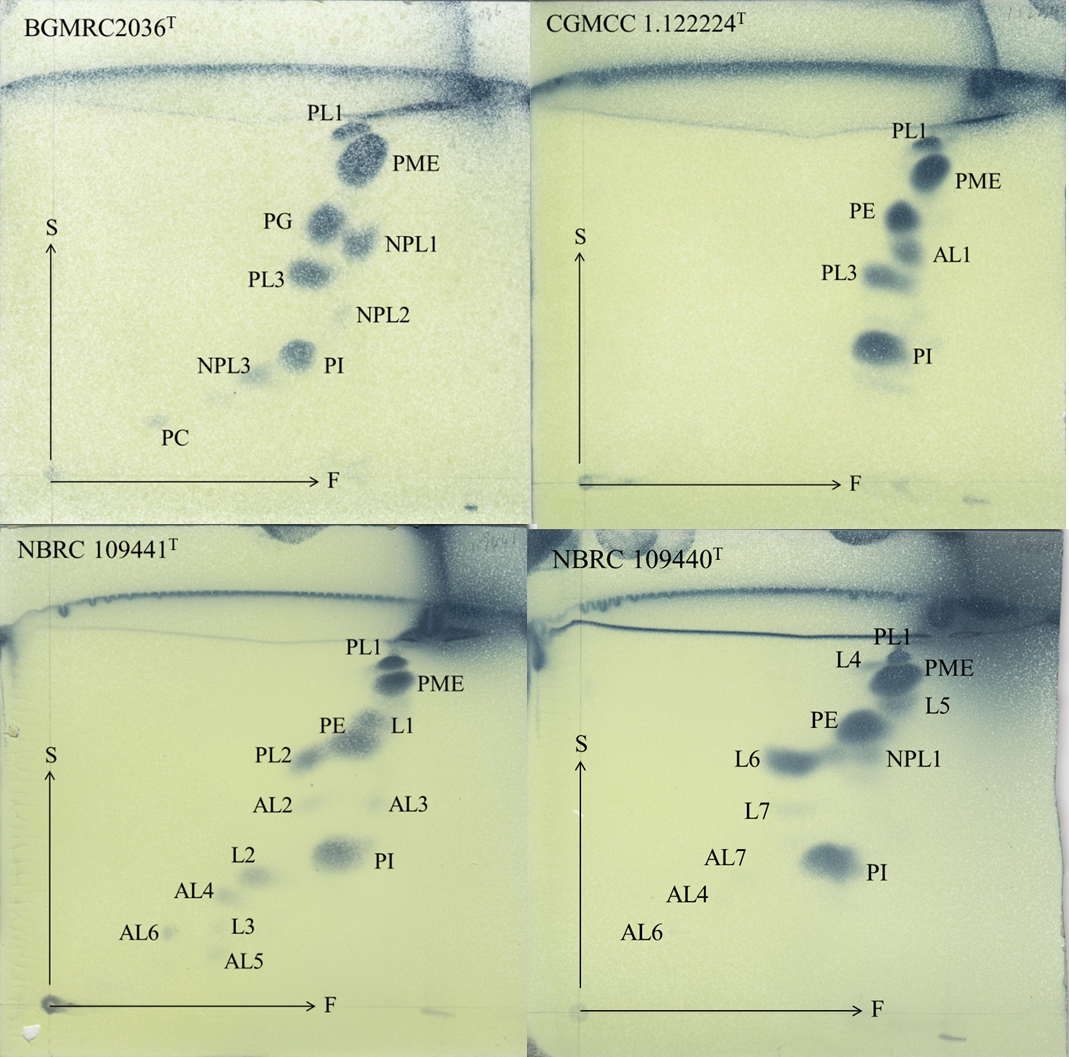
**

**Fig. S2.** Two-dimensional TLC patterns of the total polar lipids of strain BGMRC 2036^T^ and related type strains. PG, phosphatidylglycerol; PME, phosphatidylmethylethanolamine; PE, phosphatidylethanolamine; PI, phosphatidyl inositol; PC, phosphatidylcholine; AL, unidentified ninhydrin positive lipid; PL, unidentified phospholipid; NPL, unidentified ninhydrin positive phospholipid; L, unidentified lipid. F: first dimension; S: second dimension. The solvent systems used were chloroform-methanol-water (65: 25: 4, v/v) for the first dimension and chloroform-acetic acid-methanol-water (80: 15: 12: 4, v/v) for the second dimension. Phosphomolybdic acid was used to detect all lipids.

*Martelella lutilitoris* GH2-6^T^ (VCLB01000016)

*Martelella radices* BM5-7^T^ (KF560339)

*Martelella endophytica* YC6887^T^ (CP010803)

*Martelella limonii* YC7034^T^ (KR233160)

*Martelella mediterranea* DSM 17316^T^ (AQWH01000065)

*Martelella mangrovi* BM9-1^T^(KF560340)

*Martelella suaedae* NBRC109440^T^ (KR233159)

*Martelella caricis* GH2-8^T^(MG650291)

***Martelella alba* BGMRC 2036^T^(MN028527)**

*Martelella* sp strain 161492 (MH001982.1)

*Mycoplana dimorpha* IAM 13154^T^(D12786)

*Agrobacterium rosae* NCPPB 1650^T^(MF443188)

*Allorhizobium taibaishanense* ATCC 14971^T^(MKIN01000011)

*Caulobacter vibrioides* CB51^T^(AJ009957)

54

86

100

91

99

88

72

79

61

66

54

0.01

**Fig.S3.** Minimum Evolution phylogenetic tree based on the 16S rRNA gene sequences of strain BGMRC 2036^T^ and related taxa. The sequence of the *Caulobacter vibrioides* CB51^T^ were used as outgroup. Numbers at nodes indicate percentages of 1000 bootstrap resamplings; only values above 50% are shown.

*Martelella lutilitoris* GH2-6^T^ (VCLB01000016)

*Martelella radices* BM5-7^T^ (KF560339)

*Martelella radices* BM5-7^T^ (KF560339)

*Martelella limonii* YC7034^T^ (KR233160)

*Martelella mediterranea* DSM 17316^T^ (AQWH01000065)

*Martelella mangrovi* BM9-1^T^(KF560340)

*Martelella suaedae* NBRC109440^T^ (KR233159)

*Martelella caricis* GH2-8^T^(MG650291)

***Martelella alba* BGMRC 2036^T^(MN028527)**

*Martelella* sp strain 161492 (MH001982.1)

*Agrobacterium rosae* NCPPB 1650^T^(MF443188)

*Allorhizobium taibaishanense* ATCC 14971^T^(MKIN01000011)

*Mycoplana dimorpha* IAM 13154^T^(D12786)

*Caulobacter vibrioides* CB51^T^(AJ009957)

99

87

91

83

75

75

50

50

0.02

**Fig.S4.** Maximum likelihood phylogenetic tree based on the 16S rRNA gene sequences of strain BGMRC 2036^T^ and related taxa. The sequence of the *Caulobacter vibrioides* CB51^T^ were used as outgroup. Numbers at nodes indicate percentages of 1000 bootstrap replicates; only values above 50% are shown. Bar, 0.01 substitutions per nucleotide position.
